# Structure-based design and functional studies of novel noroviral 3C protease chimaeras offer insights into substrate specificity

**DOI:** 10.1042/BJ20140959

**Published:** 2014-12-05

**Authors:** Morgan R. Herod, Cynthia A. Prince, Rachel J. Skilton, Vernon K. Ward, Jonathan B. Cooper, Ian N. Clarke

**Affiliations:** * Molecular Microbiology Group, University of Southampton, Southampton SO16 6YD, U.K.; † Otago School of Medical Sciences, Department of Microbiology and Immunology, University of Otago, P.O. Box 56, Dunedin 9054, New Zealand; ‡ Laboratory for Protein Crystallography, Centre for Amyloidosis and Acute Phase Proteins, UCL Division of Medicine (Royal Free Campus), Rowland Hill Street, London NW3 2PF, U.K.

**Keywords:** murine norovirus (MNV), norovirus, protease, polyprotein, positive sense, proteolysis

## Abstract

The norovirus NS6 protease is a key target for anti-viral drug development. Noroviruses encode a 2200 amino acid polyprotein which is cleaved by this critical protease at five defined boundary substrates into six mature non-structural (NS) proteins. Studies of the human norovirus (HNV) NS6 protease, in the context of a full ORF1 polyprotein, have been severely hampered because HNVs are not culturable. Thus, investigations into the HNV NS6 protease have been largely restricted to *in vitro* assays using *Escherichia coli*-expressed, purified enzyme. The NS6 protease is formed of two distinct domains joined by a linking loop. Structural data suggest that domain 2 of the protease possesses substantial substrate binding pockets which form the bulk of the interactions with the NS boundaries and largely dictate boundary specificity and cleavage. We have constructed chimaeric murine norovirus (MNV) genomes carrying individual domains from the HNV protease and demonstrated by cell transfection that chimaeric HNV proteases have functional activity in the context of the full-length ORF1 polyprotein. Although domain 2 primarily confers boundary specificity, our data suggest that an inter-domain interaction exists within HNV NS6 protease which influences cleavage of specific substrates. The present study also shows that chimaeric MNVs provide improved models for studying HNV protein function in the context of a full ORF1 polyprotein.

## Abstract (precis)

In this study, we use a chimaeric approach to model how an intra-domain interaction between domain 1 and domain 2 of the norovirus NS6 protease influences cleavage at specific non-structural substrate boundaries.

## INTRODUCTION

Noroviruses are members of the calicivirus family of positive-sense, single-stranded RNA viruses. Human noroviruses (HNVs) are the leading cause of acute non-bacterial gastroenteritis, with epidemics common in semi-enclosed communities such as hospitals, schools and cruise ships. Currently, there is no vaccine or anti-viral therapy for treating HNV infection.

A major factor limiting the progress of vaccine and anti-viral development is the lack of a robust cell culture system for studying the molecular mechanisms of HNV infection and pathogenesis, with most recent studies using murine norovirus (MNV) which can be cultured, as a surrogate for studying HNV biology.

The norovirus genome is translated into three open reading frames (ORFs), ORF1, ORF2 and ORF3 [1–3]. ORF2 and ORF3 encode the major and minor viral structural proteins, VP1 and VP2, respectively [4,5]. ORF1 encodes a large polyprotein precursor of approximately 200 kDa which is cleaved into the six mature non-structural (NS) proteins, NS1/2-NS7, by the ORF1-encoded NS6 protease [6–15]. It is likely that all the viral NS proteins are essential for viral RNA replication, as is complete processing of the ORF1 polyprotein, making the NS6 protease an attractive target for anti-viral therapy [7,11,16,17]. Efforts to study the HNV protease *in cellulo* have been severely hampered due to the limited systems available for studying HNVs. NS6-mediated scissile bond cleavage of the ORF1 polyprotein occurs at five NS boundaries, defined by Q-G, E-G or E-A at the cleavage junctions, with the addition of Q-N in the case of the MNV [6,16–21]. Cleavage of the five NS boundaries follows a preferred temporal order, which is likely to be partially dictated by the amino acids in the P5–P2 and P2′ positions flanking the scissile bond dipeptide (P1–P1′) [6,7,16,22–25]. Comparison of the amino acid sequences flanking the scissile bonds between HNV and MNV reveal some significant diversity, suggesting differences in the temporal processing of the HNV and MNV ORF1 polyproteins.

Previous studies have reported the crystal structures of HNV and MNV proteases, either alone or in complex with natural substrate peptides and peptide-like inhibitors [13,26–29]. The norovirus NS6 protease is a cysteine protease that adopts a chymotrypsin-like fold consisting of two well-defined domains, a β-sheet domain 1 and a β-barrel domain 2, joined by the 20-residue lpeI loop. The two domains are separated by the active site groove, consisting of a catalytic triad of cysteine (Cys^139^), histidine (His^30^) and either a glutamic acid (Glu^54^) or aspartic acid (Asp^54^) residue in HNV and MNV respectively [13,23,27,28,30–32].

Specific subsites or ‘pockets’ within the protease, which interact with the substrate boundary upstream of the cleavage junction, are well defined and named S5–S1 according to the standard nomenclature for proteases [33]. Crystal structures of the protease in complex with boundary substrates show that, outside the catalytic triad, the majority of the substrate binding interactions are within domain 2 of the protease, in particular within the S2 and S4 pockets, which interact with the P2 and P4 boundary residues, respectively [26,28,29]. Upon substrate binding, the HNV protease can undergo a conformational change in the S2 and S4 pockets to accommodate variations in the P2–P5 boundary residues, and it has been suggested that this mechanism is how the HNV protease recognizes cleavage boundaries with different affinities [29]. In comparison with the S5–S1 pockets, the prime-side binding pockets, which interact with the P′ residues downstream of the cleavage junction, are less well defined. Analysis of the residues which occupy the boundary P1′ position suggest residues with smaller side-chain groups are preferred, therefore it is likely only a small S1′ pocket is required, and biochemical studies suggest the P2′ residue has only minor effects on cleavage efficiency [25]. However, there is a relative paucity of structural information on the prime-side interactions and inspection of the available crystal structures reveals that the enzyme does not appear to have a major binding groove extending beyond the S1′ pocket.

Since domain 2 of the protease contains the majority of the residues which form the S5–S1 pockets and interact with the cleavage boundary, we hypothesized that domain 2 dominates boundary specificity to dictate cleavage of the ORF1 polyprotein. To investigate this hypothesis, we made chimaeric MNV constructs by exchanging individual domains from the MNV NS6 protease, or the protease in its entirety, with the equivalent portion from the HNV protease. In doing so, we demonstrate that chimaeric MNV/HNV proteases show functional activity and that the HNV protease is able to process the MNV ORF1 polyprotein. In addition, results presented in the present study suggest that although domain 2 may confer the majority of the boundary specificity, an inter-domain interaction within HNV NS6 influences cleavage at specific NS boundaries. Furthermore, we propose that these ‘humanized’ MNV constructs, which carry a HNV NS6 protease in place of the MNV protease, provide an additional model for further understanding of HNV protease function *in cellulo*, within the context of a full ORF1 polyprotein.

## MATERIALS AND METHODS

### Plasmid construction

The polII based MNV plasmid expression construct, pMNV*, from which all pMNV* mutants were derived, has been described previously [34]. The sequence of all the primers used in the present study can be found in Supplementary Table S1. Introduction of the GDD>GAD polymerase inactivating mutation was performed by PCR using primers GAD_Fwd and GAD_Rvs and the subsequent AfeI–XhoI fragment was cloned into AfeI–XhoI-digested pMNV*.

To introduce the entire Southampton virus (SV) NS6 sequence mutagenic PCR was used, with first round amplification using primers SV3C_Fwd + SVD2_MNV3CDP6-P2′_Rvs with template pT7-SV3BCD (shown in Figure S1 and described below) [35], to amplify the entire SV NS6 fragment with flanking MNV* sequence. First round PCRs to amplify the upstream and downstream MNV* sequence were performed using primers MNV_4F + SV3C_Rvs and primers SVD2_MNV3CDP6P2′_Fwd + MNV_6R, with template pMNV*. The main PCR product from the second round of amplification, generated from products of the first round in combination with primers MNV_4F and MNV_6R, was digested with AflII and cloned into AflII-digested pMNV* to make pMNV*-SV3C.

Generation of the domain swap mutants was performed by standard overlapping 2-step PCR mutagenesis [36]. Primers MNV_SV3CD1_Fwd + MNV_6R and MNV_SV3CD1_Rvs + MNV_4F were used in the first round amplification with templates pMNV* and pMNV*-SV3C, respectively. The first round PCR products were used in the second round of amplification with primers MNV_4F and MNV_6R and the AflII-digested second round PCR product cloned into AflII-digested pMNV*. The domain 2 swap was generated using a similar cloning strategy involving primers MNV_SV3CD2_Fwd + MNV_6R and MNV SV3CD2_Rvs + MNV_4F in the first round reactions with templates pMNV*-SV3C and pMNV*, respectively.

The AK29TT and D54E coding changes were introduced using standard two-step PCR mutagenesis, with first round reactions using primers MNV3CD1_AK29TT_Fwd or MNV3CD1_D54E_Fwd with MNV_6R and MNV3CD1_AK29TT_Rvs or MNV3CD1_D54E_Rvs with MNV_4F, with template pMNV*-SV3CD1. The second round PCR products, generated using the products from the first round and primers MNV_4F + MNV_6R, were digested with AflII and cloned into AflII-digested pMNV*. The same strategy was used to introduce the TT29AK and E54D mutations into pMNV*-SV3CD2 with mutagenic PCR primers MNVSV3CD1_TT29AK_Fwd + MNVSV3CD1_TT29AK_Rvs and MNVSV3CD1_E54D_Fwd + MNVSV3CD1_E54D_Rvs.

The T7-based *Escherichia coli* expression construct, pSV3C (pT7-7/SV9), referred to in the present study as pT7-SV3BCD, has been previously described [35]. Briefly, PCR mutagenesis was used on template plasmid pSVFrag2 [19] to amplify the SV NS6 coding region including the surrounding 24 bp of upstream and 444 bp of downstream SV sequence, and introduce an unique upstream NdeI and downstream BamHI restriction enzyme site. The subsequent PCR product was digested with NdeI and BamHI and introduced into NdeI–BamHI-digested pT7-7 (USB Corp.) to make pT7-SV3BCD (map and sequence given in Figure S1).

The similar MNV construct, pRSETA-MNV3BCD, was generated by PCR, using primers NS6_Fwd + NS6_Rvs with template pMNV*, to amplify the entire NS6 sequence including the flanking 123 bp of MNV* sequence at both ends. The amplified PCR product was digested with BamHI and EcoRI and introduced into the expression vector pRSETA (Invitrogen) at the BamHI and EcoRI sites. For generating the pRSETA-derived constructs expressing the domain swap chimaeric proteases, the unique SalI–Bsu36I fragment from pRSETA-MNV3BCD, which covers the entire protease gene and upstream/downstream flanking regions, was exchanged with the equivalent SalI–Bsu36I fragment from the pMNV* domain swap vectors.

### Expression and purification of NS6 protease in *E. coli*

*E. coli* overexpression of pRSETA based constructs and subsequent protease purification was carried out as previously described [26]. Following the final desalting step the protein was concentrated to approximately 5 mg/ml and stored in 50% glycerol.

### *In vitro* kinetic assay for protease activity

Kinetic studies on purified NS6 proteases were performed as previously described [26], using the synthetic peptide Ac-DEFQLQ-*p*NA (Peptide Protein Research) which contains an acetylated (Ac) N-terminus and a C-terminal *p*-nitroaniline (*p*NA) group to provide a spectrophotometric output. The rate of cleavage of this substrate (final concentration 0.1–4 mM) by NS6 protease at a final concentration of 0.1 mg/ml was monitored at 415 nm using a iMark plate reader (Bio-Rad) over a 10 min time period. Results show mean kinetic values of three independent experiments.

### SDS/PAGE and Western blot analysis

SDS/PAGE and Western blot were carried out as previously described [34]. Primary antibodies to MNV NS proteins used were rabbit anti-NS4, rabbit anti-NS6, rabbit anti-NS7, and mouse anti-NS1/2 [37], mouse anti-NS3 and mouse anti-NS7 [38]. Primary antibody to SV NS6 is a mouse monoclonal antibody which only detects SV NS6 domain 1 and does not cross react with MNV NS6. Goat anti-rabbit and goat anti-mouse horseradish peroxidase conjugates (Sigma–Aldrich) were used as secondary reagents. Densitometry was conducted using ImageJ imaging analysis software on duplicate experiments. Data are means with S.D.

### Cell culture and transfection

HEK-293T cells (obtained from the A.T.C.C., LGC Standard) were grown in Dulbecco's modified Eagle's medium supplemented with 10% FBS and GlutaMAX-1 (Invitrogen). Transfection of HEK-293T cells was performed as previously described [34] using 1 μg of pMNV* plasmid DNA and 1 μg of ptTA per well.

### Computer modelling of the chimaeric domain swapped protease structures

Models of the three-dimensional structures of the two domain swapped chimaeric proteases were produced by least-squares superposition of the SV and MNV protease structures [26,28]. Following this superposition, the two domains of each protease were inter-changed and the resulting models were checked for steric hindrance using the program CONTACT [39,40]. Minor remodelling where necessary to relieve local poor contacts was conducted using the graphics program COOT [41]. The theoretical energetics of the resulting domain interfaces were analysed using the PISA server [42].

## RESULTS

### Chimaeric NS6 proteases demonstrate proteolytic activity in *E. coli*

The structure of MNV and HNV NS6 protease in complex with boundary substrates and substrate-like inhibitors strongly suggests that domain 2 is primarily responsible for recognizing all five NS protein boundaries and dictating cleavage specificity [26,28,29]. The aim of the present study was to investigate whether domain 2 of the protease alone dictates boundary recognition and cleavage by exchanging individual domains from the MNV protease with the equivalent domain from the HNV SV protease to construct chimaeric NS6 proteases. Our working hypothesis was that by exchanging just domain 2 of the protease, it should be possible to replicate the cleavage specificity observed by exchanging both domains of the protease.

**Figure 1:**
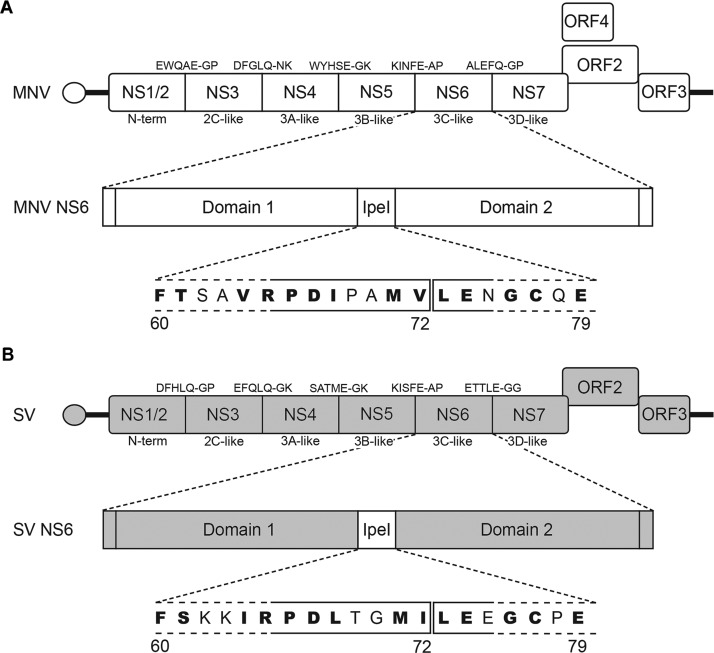
Schematic representation of the MNV and SV genomes The genomes of murine norovirus 1 (**A**) and the human norovirus strain SV (**B**) are aligned and annotated with the P5–P2′ residues of the ORF1 cleavage sites and a schematic representation of the NS6 protease with the amino acid sequence found within the lpeI loop. Sequence similarity between the two lpeI loops is indicated in bold. The position where the protease domains were swapped is indicated on the lpeI sequence.

**Figure 2:**
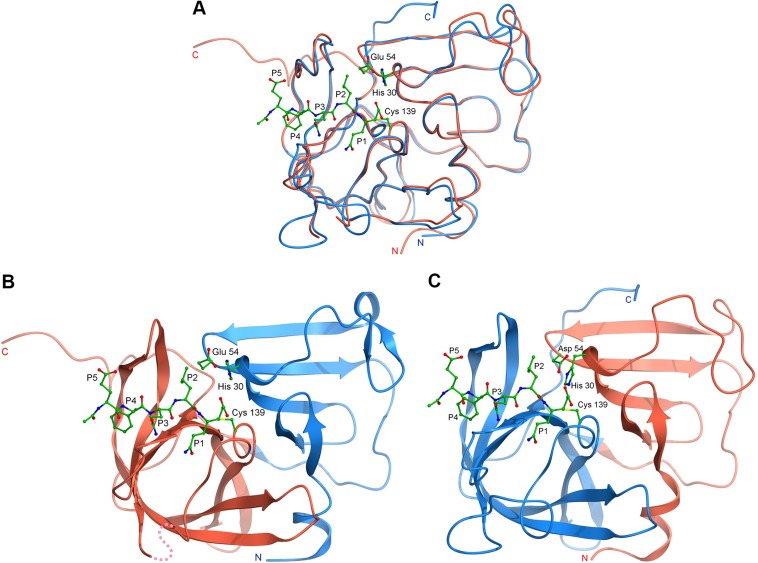
Computer modelling of the domain swap chimaeric norovirus protease (**A**) Model showing a superposition of the SV and MNV protease structures in blue and red, respectively, with a HNV substrate-like inhibitor and catalytic triad drawn in ball-and-stick representation. Both structures suggest that domain 2 of the protease (shown on the left of diagram) is responsible for the majority of the boundary interactions. (**B**) Computer model of the chimaeric protease in which domain 1 of the SV NS6 protease (blue) has been fused to domain 2 of the MNV protease (red). (**C**) The converse chimaera in which domain 2 of the SV NS6 protease (blue) is fused with domain 1 of the MNV enzyme (red).

Alignment of the MNV and SV NS6 20-residue lpeI loop, which connects the two protease domains, shows that the greatest sequence identity exists between Met^71^ and Cys^77^. It was therefore decided to splice the domains within the lpeI loop between residues 72 and 73 (Figure 1). Computer modelling of domain swap proteases by superposition of the SV and MNV protease structures [26,28], suggested that the heterologous domains of MNV and SV NS6 would ‘fit’ together well, indicating that a chimaeric domain swapped protease was likely to retain proteolytic activity (Figure 2). Indeed, the two domains of the chimaeric protease carrying SV domain 1 have a very favourable predicted interface energy (Δ*G*=−21.8 kcal/mol; 1 kcal=4.184 kJ) and are predicted to form a total of seven hydrogen bonds and three salt bridges. Likewise, the two domains of the chimaeric protease carrying SV domain 2, are estimated to interact with a similar Δ*G* (−22.3 kcal/mol) and form 11 hydrogen bonds and five salt bridges. Both of these values compare very favourably with the equivalent values of −19.5 kcal/mol (17 hydrogen bond and 12 salt bridges) for the two domains of the wild-type SV protease and −21.1 kcal/mol (six hydrogen bonds and two salt bridges) for the wild-type MNV protease, strongly suggesting the chimaeric enzymes are likely to be as stable as the wild-type proteases.

**Figure 3:**
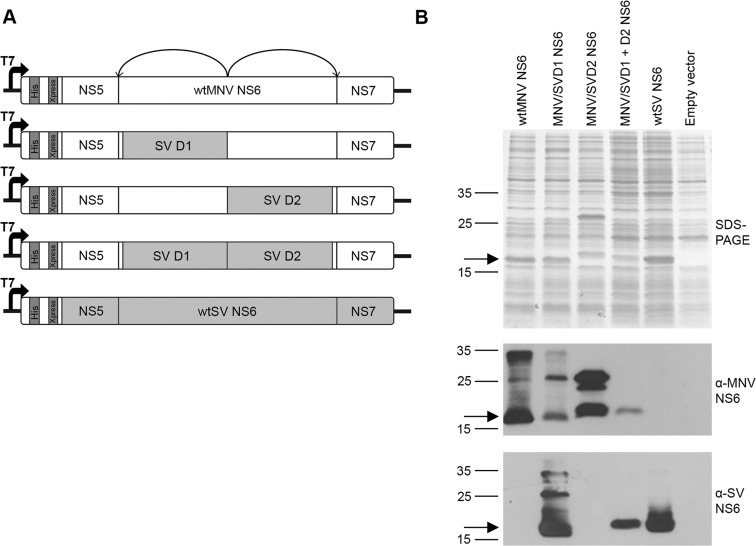
Analysis of heterologously expressed NS6 domain swap proteases (**A**) Schematic representation of the domain swap constructs used for *in vitro* expression. Unshaded boxes represent wild-type (wt) MNV sequence, whereas light-grey boxes represent wild-type (wt) SV sequence. The dark grey sequence shows the His tag and Xpress epitope. Curly arrows indicate the positions where the NS6 protease can self excise from the ‘mini’ NS5–NS7 precursor. (**B**) SDS-PAGE and Western blot analysis of the expressed chimaeric NS6 proteases. Induced *E. coli* lysates were analysed by SDS-PAGE and Western blot using antibodies specific for MNV NS6 or SV NS6. Arrows indicates the position of fully cleaved NS6 proteases which is consistent with their predicted molecular masses. The bands at approximately 27, 23 and 33 kDa correspond to the predicted molecular masses of the uncleaved ‘mini’ NS5NS6, NS6NS7 and NS5–NS7 precursors, respectively.

We first sought to confirm experimentally that heterologously expressed chimaeric protease would have proteolytic activity on MNV cleavage boundaries using an *E. coli* expression system. This system, developed in our laboratory using wild-type SV protease, uses a T7 vector to express MNV NS6 flanked by 41 residues of upstream NS5 sequence and 41 residues of downstream NS7 sequence to produce a ‘mini’ NS5–NS7 precursor. Fused upstream of the NS5 sequence are 36 amino acids of vector sequence containing a His tag and Xpress epitope (Figure 3A). Once expressed in *E. coli*, the active protease is able to cleave the upstream NS5NS6 and downstream NS6NS7 boundaries, releasing the 19 kDa mature NS6. This system was subsequently modified to introduce either SV domain 1, SV domain 2 or both SV domain 1 and domain 2 in a double domain swap, in place of the equivalent MNV sequence, and the production of fully cleaved NS6 was analysed by SDS-PAGE and Western blot (Figure 3B).

For both MNV and SV wild-type proteases and all three chimaeric domain swap proteases, a mature NS6 protease band was clearly observed by SDS-PAGE and confirmed by Western blot. The full-length SV protease, in both the wild-type context and with flanking MNV sequence, self-excised completely from the precursor (Figure 3B, third panel, lanes 4 and 5). However, the wild-type MNV protease (Figure 3B, second panel, lane 1) and the two chimaeric domain swap proteases (Figure 3B, second panel, lanes 2 and 3) showed some residual uncleaved precursors, as confirmed by Western blot with an NS7 antiserum (Figure S2). These data confirm that all the chimaeric domain swap proteases have proteolytic activity on MNV cleavage boundaries, despite the fact there is some variation in the cleavage of these boundary substrates, based on the relative amount of residual precursors remaining. This is most notable when comparing the wild-type MNV protease with the double domain swap protease and wild-type SV protease, both of which were fully able to cleave themselves out of the precursor, demonstrating the wild-type MNV protease is less active on these boundaries in this assay.

### *In vitro* kinetic analysis of purified chimaeric NS6 proteases

Having established that all the chimaeric NS6 proteases were able to self-excise from the ‘mini’ NS5–NS7 precursor to yield mature NS6, we were able to produce highly purified protease from all five constructs and conduct a comparative kinetic analysis. The five NS6 proteases were purified from whole *E. coli* lysates as previously described and analysed by non-reducing PAGE (Figure S3) [26]. A major band was observed at approximately 18 kDa for all of the purified enzymes. With the exception of the chimaeric protease carrying SV domain 2 (MNV/SVD2 NS6), an additional minor band was observed at approximately 36 kDa which corresponds to the dimeric form of the NS6 protease as has previously been observed [13,28,43,44].

Measurements of the initial rate of cleavage were performed for each chimaeric protease (and wild-type controls) over a 10 min time period using the chromogenic *p*NA peptide Ac-DEFQLQ-*p*NA, a synthetic substrate representing the SV NS3NS4 cleavage boundary (Table 1 and Supplementary Figures S4A and S4B). Little difference was observed in the activity of the wild-type SV and MNV proteases in terms of the *k*_cat_ or *K*_m_ and thus minimal variation was observed in activity in terms of their specificity constant (*k*_cat_/*K*_m_). Compared with wild-type protease, the double domain swap chimaera (MNV/SVD1+D2 NS6) demonstrated a less than 2-fold reduction in activity in terms of the *k*_cat_ and a minor reduction in *K*_m_, resulting in overall a less than 2-fold reduction in activity in terms of specificity constant (*k*_cat_/*K*_m_). The chimaeric protease carrying SV domain 1 (MNV/SVD1 NS6) also showed a reduction in *k*_cat_ of approximately 3-fold compared with the wild-type proteases. However, a less than 2-fold reduction in *K*_m_ resulted in only a 2-fold overall reduction in activity in terms of the specificity constant. In contrast, the chimaeric protease carrying SV domain 2 (MNV/SVD2 NS6) demonstrated no proteolytic activity on this substrate during the assay period. Furthermore, increasing the assay period for up to 4 h resulted in no increase in OD above background levels for this enzyme (Supplementary Figure S4C).

**Table 1:** Kinetic data for the hydrolysis of the substrate DEFQLQ-*p*NA by chimaeric NS6 proteases wt, wild-type.

Enzyme	*K*_m_ (M)	*k*_cat_ (s^−1^)	*k*_cat_/*K*_m_ (M^−1^·s^−1^)
wtMNV NS6	2.33×10^−3^	0.08	34
MNV/SVD1 NS6	1.53×10^−3^	0.03	19.6
MNV/SVD2 NS6	No activity	No activity	No activity
MNV/SVD1+D2 NS6	2.16×10^−3^	0.06	28
wtSV NS6	2.82×10^−3^	0.11	39

### Chimaeric NS6 proteases have proteolytic activity in MNV

**Figure 4:**
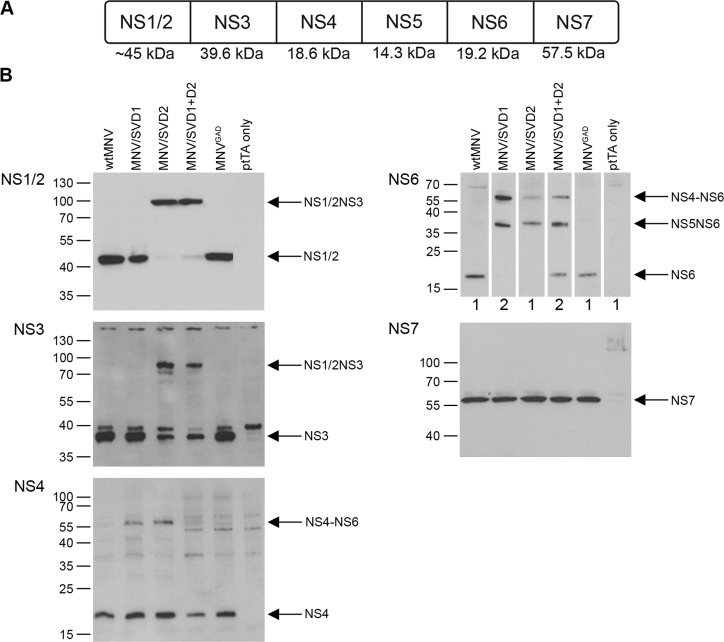
Analysis of NS protein expression in full-length MNV* constructs containing the chimaeric domain swap mutations (**A**) Schematic layout of the MNV ORF1 polyprotein with the molecular mass of each NS protein indicated. (**B**) HEK-293T cells were transfected with full-length wild-type (wt) MNV*, constructs carrying the chimaeric NS6 proteases (MNV/SVD1, MNV/SVD2 and MNV/SVD1 + D2) or a polymerase knockout construct (MNV^GAD^), under control of a tetracycline-responsive CMV (cytomegalovirus) promoter. Maximal promoter activity requires co-transfection with ptTA, a plasmid expressing the tetracycline trans-activator. Control lysate was derived from cells transfected with ptTA only. Cell lysates were harvested at 24 h post-transfection and probed by Western blot for expression of MNV proteins (NS1/2, NS3, NS4, NS6 and NS7) and SV NS6. The image for NS6 shows a composite for both anti-MNV NS6 (1) and anti-SV NS6 (2) Western blots. Arrows indicate the positions of full-length proteins and different polyprotein precursors. Note the anti-NS3 antibody detects a cross-reactive band at approximately 40 kDa which can be clearly seen in the negative control lane.

The above data showed that heterologously expressed domain 1 and double domain swap chimaeric proteases have proteolytic function on both the MNV NS5NS6 and NS6NS7 boundaries, and on an SV cleavage peptide. Furthermore, although the chimaeric protease carrying SV domain 2 showed functional activity in *E. coli* on the NS5NS6 and NS6NS7 MNV boundaries, no functional activity was detected on the synthetic substrate representing the SV NS3NS4 cleavage boundary, suggesting this enzyme has restricted boundary recognition and therefore limited proteolysis. However, the *in vitro* kinetic analysis was limited to a single synthetic boundary outside the context of a full ORF1 polyprotein and not in the context of a eukaryotic background. Therefore, to investigate any potential variations in boundary proteolysis in a full-length ORF1 polyprotein, where all five MNV cleavage boundaries are present, all three chimaeric proteases were introduced into MNV*, a construct expressing the entire MNV cDNA under control of a tTA responsive polII promoter. Transfection of MNV* constructs into permissive cell lines allows for export of capped viral transcripts to the cell cytoplasm and expression of the full ORF1 polyprotein where cleavage of the NS boundaries can be easily accessed by Western blot. The chimaeric MNV* constructs and controls were co-transfected into HEK-293T cells with ptTA, a plasmid expressing the tetracycline trans-activator allowing for maximal promoter activity, and NS protein cleavage was assayed by Western blot (Figure 4). In HNV it is hypothesized that the NS1/2NS3 and NS3NS4 boundaries are the first to be processed, both of which have glutamine residues at the P1 position [6,16,22]. Therefore, we may minimally anticipate successful cleavage at the MNV NS3NS4 and NS6NS7 boundaries, both of which have glutamine in the P1 position.

Consistent with this prediction, all chimaeric proteases demonstrated complete cleavage at the MNV NS3NS4 and NS6NS7 boundaries which have glutamine residues at the P1 position. Only the chimaeric construct carrying SV domain 1 (MNV/SVD1) showed complete processing at the NS1/2NS3 boundary, yielding mature NS1/2 and NS3. Both chimaeric constructs carrying either SV domain 1 or SV domain 2 (MNV/SVD1 and MNV/SVD2, respectively) demonstrated little or no processing at the NS4NS5 and NS5NS6 boundaries, yielding mainly uncleaved NS4–NS6 and NS5NS6 precursors and a moderate amount of fully cleaved NS4. Interestingly, the double domain swap chimaera (MNV/SVD1 + D2) showed partial processing at both the NS4NS5 and NS5NS6 boundaries, resulting in a moderate amount of mature NS4 and NS6. Due to the unavailability of an anti-NS5 antibody capable of detecting the relatively low abundance of protein in this system, assignment of NS5 containing precursors was done by elimination.

### Residues within NS6 domain 1 facilitate cleavage at the NS5NS6 boundary

Our original hypothesis was that domain 2 alone interacts with the boundary sequence to define cleavage specificity. Thus, the cleavage profile observed by exchanging solely domain 2 should be the same as exchanging both domains of the protease. Contrary to this expectation, although the double domain swap chimaera demonstrated partial cleavage at the NS5NS6 boundary, the chimaeric protease carrying SV domain 2 showed a complete block in NS5NS6 processing. This suggests that boundary specificity is not conferred by domain 2 alone, and domain 1 must contribute some key residues which influence cleavage at certain NS boundaries.

**Figure 5:**
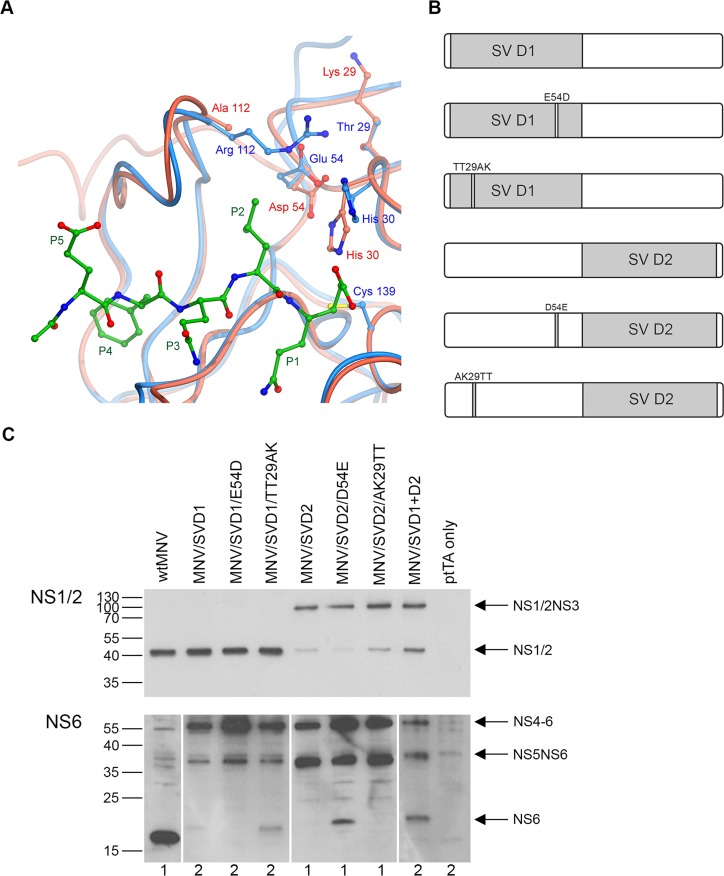
Analysis of NS protein expression in chimaeric MNV* constructs carrying additional domain 1 mutations (**A**) A superposition of the MNV protease (red) and SV protease (blue) structures indicating the residues which were chosen for mutagenesis. An example cleavage substrate bound to the SV protease is shown in green. (**B**) Schematic representation of the chimaeric domain swap proteases carrying additional domain 1 mutations that were introduced into full-length MNV*. Unshaded boxes indicate MNV sequence, whereas light-grey shaded boxes represent SV sequence. (**C**) HEK-293T cells were transfected with full-length wild-type (wt) MNV*, constructs carrying the chimaeric NS6 proteases or chimaeric constructs carrying domain 1 additional mutations. Cells were co-transfected with ptTA for maximal promoter activity. Control lysate was derived from cells transfected with ptTA only. Cell lysates were harvested at 24 h post-transfection and probed by Western blot for expression of MNV NS1/2, MVN NS6 and SV NS6. The image for NS6 shows a composite for both MNV NS6 (1) and SV NS6 (2) Western blots. Arrows indicate the positions of full-length proteins and different polyprotein precursors.

Potentially a key difference between HNV and MNV proteases is the change from a glutamic acid to aspartic acid within the catalytic triad, at position 54 within domain 1 of the enzyme (Figure 5A). Comparison of the domain 1 residues surrounding the active site between HNV and MNV showed a further difference in the residues at positions 28 and 29 (adjacent to His^30^ of the catalytic triad), which are changed from two threonines in SV protease to an alanine and a lysine in the MNV protease (Figure 5A). To investigate whether these differences in domain 1 could affect boundary recognition and cleavage, PCR mutagenesis was used to introduce a D54E or AK29TT coding change within the MNV domain 1 sequence of the chimaeric MNV* construct carrying SV domain 2 (Figure 5B). Such changes represent the residues which are found at these positions within wild-type SV protease. In parallel, E54D and TT29AK coding changes were introduced into the SV domain 1 sequence of the chimaeric MNV* construct carrying SV domain 1. These changes represent the residues that are found at these positions within the wild-type MNV protease (Figure 5B). NS1/2NS3 and NS4–NS6 boundary cleavage in these new MNV* constructs was analysed by Western blot using antibodies against NS1/2, MNV NS6 and SV NS6 (Figure 5C).

The chimaeric construct carrying SV domain 1 showed complete processing at all NS boundaries assayed except the NS4NS5 and NS5NS6 boundaries, resulting in an accumulation of uncleaved NS4–NS6 and NS5NS6 precursors. Introducing the E54D mutation into this construct (MNV/SVD1/E54D) had no effect on NS boundary cleavage. However, introduction of the TT29AK mutations (MNV/SVD1/TT29AK) moderately increased cleavage at the NS5NS6 boundary yielding a limited amount of mature NS6 protease. The chimaeric construct carrying the SV domain 2 showed a limitation in cleavage at the NS4NS5 and NS5NS6 boundaries (yielding approximately 37±3% NS4-6 and 62±3% NS5NS6 by densitometry), in addition to limited processing of the NS1/2NS3 boundary. Introduction of the AK29TT mutations within MNV domain 1 of this construct (MNV/SVD2/AK29TT) resulted in no change in NS boundary cleavage (approximately 38±0.5% NS4–NS6 and 62±1% NS5NS6 by densitometry). However, mutation of the aspartic acid residue at position 54 to a glutamic acid (MNV/SVD2/D54E) resulted in increased cleavage at the NS5NS6 boundary, yielding mature NS6 to levels similar to that observed in the double domain swap chimaeric construct (11±5% compared with 10±7% for MNV/DV2D/D54E and MNV/SVD2, respectively). Although it is worth noting that this D54E mutant protease (MNV/SVD2/D54E) still demonstrated a greater proportion of uncleaved NS4-6 and less NS5NS6 (49%±0.2 and 39%±6 of NS4-6 and NS5NS6, respectively) when compared with the double domain swap chimaeric construct (3%±0.4 and 87%±6 of NS4-6 and NS5NS6, respectively).

### Glu^54^ in domain 1 restores minimal kinetic activity of the domain 2 chimaeric enzyme

Introduction of the TT29AK mutation into SV domain 1 of the chimaeric MNV construct carrying SV domain 1 increased cleavage at the NS5NS6 boundary, resulting in the release of some mature NS6. Likewise, introduction of the D54E mutation into MNV domain 1 of the chimaeric construct carrying SV domain 2 also resulted in increased cleavage at the NS5NS6 boundary, yielding mature NS6. To examine if such mutations affected enzyme kinetics, the ‘mini’ NS5–NS7 precursor system described above was employed to express the chimaeric SV domain 1 NS6 carrying the TT29AK mutation and the chimaeric SV domain 2 NS6 carrying the D54E mutation (results not shown). Purified protease was produced from whole *E. coli* lysates (results not shown), and measurements of the initial rate of cleavage performed, as before, with wild-type MNV and SV proteases included as internal controls (Table 2).

**Table 2:** Kinetic data for the hydrolysis of the substrate DEFQLQ-*p*NA by chimaeric NS6 proteases containing additional domain 1 mutations wt, wild-type

Enzyme	*K*_m_ (M)	*k*_cat_ (s^−1^)	*k*_cat_/*K*_m_ (M^−1^·s^−1^)
wtMNV NS6	1.0×10^−3^	0.053	49
MNV/SVD1/TT29AK NS6	1.65×10^−3^	0.0052	3.2
MNV/SVD2/D54E NS6	2.75×10^−3^	0.0018	0.7
wtSV NS6	0.7×10^−3^	0.053	76

Little difference was observed in the kinetic activities of the wild-type MNV or SV NS6 proteases on this substrate which showed *k*_cat_/*K*_m_ values not significantly different to those observed in Table 1, demonstrating the reproducibility of this assay. The TT29AK mutation in the chimaeric NS6 carrying SV domain 1 (MNV/SVD1/TT29AK NS6) resulted in approximately a 10-fold reduction in *k*_cat_ when compared with either wild-type protease, and as a result showed a 10-fold reduction in the specificity constant (*k*_cat_/*K*_m_). Conversely, introduction of the D54E mutation within the chimaeric NS6 carrying SV domain 2 (MNV/SVD2/D54E NS6), restored a minimal level of proteolytic activity on this substrate, albeit with a 2.5-fold greater *K*_m_ and a 30-fold reduction in *k*_cat_, resulting in approximately a 70-fold reduced specificity constant (*k*_cat_/*K*_m_) when compared with wild-type protease.

## DISCUSSION

Release of the NS proteins from the ORF1 polyprotein in noroviruses is facilitated by the NS6 protease, which cleaves at five defined scissile bond dipeptides to yield six mature NS proteins [6,7,16,17]. The two distinct domains of the norovirus NS6 protease are separated by the active site groove and linked by the conserved 20-residue hinge-like lpeI loop [13,26–29]. The residues which form the active site catalytic triad, and the S5–S1 binding pockets, have been well characterized for both HNV and MNV protease. For both enzymes, Cys^139^ has been identified as the active site nucleophile and His^30^ as the general base catalyst which is protonated on the imidazole ring. The acidic third residue of the triad, Glu^54^ in HNV and Asp^54^ in MNV is suggested to hydrogen bond with His^30^ to orientate and stabilize the imidazole ring. The S1, S2 and S4 binding pockets appear to be the most critical for substrate binding, the S2 and S4 subsites both forming particularly deep hydrophobic pockets which overlap and share residues [13,26–28,31,32].

We sought to investigate whether individual protease domains conferred specificity to cleavage sites within the MNV ORF1 polyprotein. Our initial hypothesis was that domain 2 of the protease was the main contributor to substrate specificity, dictating boundary cleavage. Accordingly, we constructed simple domain swap chimaeric proteases and re-examined polyprotein processing. First, it was surprising that proteolytic activity is retained with all the chimaeric domain swaps, considering the evolutionary distance between MNV and HNV [45] and the differences in the structures of the NS6 proteases [26,28]. Both the chimaeric constructs carrying SV domain 1 or SV domain 2 were able to process the NS3NS4, NS4NS5 and NS6NS7 boundaries to yield mature NS4 and NS7. In addition, chimaeric NS6 carrying SV domain 1 was able to process the NS1/2NS3 boundary to yield mature NS1/2 and NS3. Upon insertion of the entire SV protease, in a double domain swap chimaera, the ORF1 polyprotein was processed at the NS3NS4, NS4NS5 and NS6NS7 boundaries, additionally, partial cleavage was observed at the NS5NS6 boundary, yielding moderate amounts of mature NS5 and NS6. Contrary to our initial hypothesis, our experimental data showed that the chimaeric protease carrying SV domain 2 demonstrates restricted proteolytic activity compared with the double domain swap protease, in both *in vitro* assays and in the context of a full-length ORF1 polyprotein. These observations led to our second hypothesis, that domain 1 contributes critical residues which facilitate cleavage of specific NS protein boundaries.

Comparison of the domain 1 residues in MNV and HNV that are predicted to lie close to the boundary substrate and active site highlighted two strong potential candidate residues that could influence boundary cleavage. First, the acidic third residue of the catalytic triad is different between MNV and HNV. Secondly, a triple neutral-polar stretch of TTT at positions 27–29, just prior to His^30^, in HNV is changed to TAK in MNV. Furthermore, both Glu^54^ and the triple neutral-polar stretch are conserved across all HNV sequences (the latter being either TTT or TST depending on isolate). We therefore hypothesized that some of these differences, within domain 1 of the protease, could influence cleavage at some NS protein boundaries. In agreement with these predictions, we found that introduction of a TT29AK mutation within SV domain 1 of the chimaeric protease carrying MNV domain 2, increased cleavage at the NS5NS6 boundary, despite showing a 10-fold reduction in kinetic activity *in vitro*. Moreover, the D54E mutation within MNV domain 1 of the chimaeric protease carrying SV domain 2, restored cleavage at the NS5NS6 boundary to levels equivalent to the double domain swap chimaera, and restored minimal activity *in vitro* on the NS3NS4 substrate.

The X-ray structure of wild-type HNV protease shows that Glu^54^ within domain 1 can salt-bridge with Arg^112^ within the flexible lpbII loop of domain 2. Furthermore, computer modelling suggests that this interaction would be weaker in the chimaeric protease carrying SV domain 2 (which contains MNV domain 1 and thus Asp^54^) due to a slightly shorter side chain. Although it should be noted that the interaction between Glu^54^ and Arg^112^ is somewhat variable in the available crystal structures, and indeed the structure in which these residues come the closest together has a partially disrupted catalytic triad, it is conceivable that the Glu^54^–Arg^112^ interaction is of some physiological importance, not least because this particular glutamate is the closest negatively charged group to the guanidinium of Arg^112^. It would also be anticipated that re-introduction of Glu^54^ into MNV domain 1 of the chimaeric protease carrying SV domain 2, might strengthen this salt bridge interaction. It is possible that this salt bridge interaction is important for proteolysis of particular substrates, such as the NS5NS6 boundary (KINFE-AP). Recently, Muhaxhiri et al. [29] have observed that the HNV NS6 protease can undergo structural variation, particularly in the S2 pocket where the protease adopts a more ‘open’ conformation to accommodate bulkier P2 residues (such as the phenylalanine in KINFE-AP). Taking these observations into account, and the suggestion that the Glu^54^–Arg^112^ salt bridge interaction is required for HNV NS6-mediated cleavage of KINFE-AP substrates, it is conceivable that the Glu^54^–Arg^112^ salt bridge interaction is key in stabilizing the more ‘open’ protease structure to allow cleavage of boundaries with larger P2 residues when glutamic acid is found in the P1 position. Previous reports have shown that mutation of the acidic Glu^54^ to a basic residue (lysine or arginine) is greatly detrimental to protease stability, and introduction of many hydrophobic residues at this position restricts cleavage at certain substrate boundaries [32]. However, such restriction was generally observed at boundaries with small residues at the P2 and P4 positions (such as TATSE-GK and TTTLE-GK and not KLSFE-AP). In contradiction to the observations reported here, the same series of studies demonstrated that mutation of the Glu^54^ residue to several alternatives, including aspartic acid, had no effect on processing of the NS5NS6 boundary, despite showing a 4–5-fold reduction in kinetic activity on a NS3NS4 boundary substrate [23,32]. This discrepancy may be explained by the fact that these *in vitro* studies were conducted outside the context of the full-length polyprotein, where secondary factors such as protein folding intermediates are likely to play a role in substrate proteolysis. This is further highlighted by the observation that although the purified NS6 carrying SV domain 2 showed no kinetic activity *in vitro*, on a synthetic SV NS3NS4 boundary peptide, it was able to cleave the MNV NS3NS4 boundary when in the context of a full ORF1 polyprotein. Comparison of the SV and MNV NS3NS4 boundaries show little difference in their sequences, being EFQLQ-GK and DFGLQ-NK, respectively. It is, however, possible that the difference in the residues at the P3 and P1′ position of these boundaries may account for this difference in cleavage, given the characterized interactions between domain 2 of HNV and the P3 residue [25]. Taken together these data illustrate a clear deficiency in using *in vitro* assays, such as the use of synthetic boundary peptides with purified proteases, to draw conclusions about eukaryotic virus biology.

All of the chimaeric constructs were able to efficiently process the NS3NS4 and NS6NS7 boundaries to completion. This suggests a preference of both enzymes to cleave substrates with glutamine in the P1 position, even though structural studies suggest that the protease makes the same interactions with either glutamine or glutamic acid in the P1 position [26]. Despite this, none of the chimaeric proteases, or the double domain swap protease, were able to fully process the MNV ORF1 polyprotein to completion. All of the chimaeric constructs were only able to partially process the NS4NS5 boundary, and in addition all the constructs carrying SV domain 2, including the double domain swap construct, were unable to efficiently process the NS1/2NS3 boundary. The residues at the P2 and P4 position within these two boundaries for MNV (tryptophan and alanine for NS1/2NS3 and tyrosine and serine for NS4NS5, respectively) are not found at these positions within any SV cleavage boundary. It is therefore feasible that these residues render these boundaries partially refractory to cleavage by the SV protease. Furthermore, it would superficially appear that the NS5NS6 boundary is the most conserved cleavage boundary between SV and MNV, being KISFE-AP and KINFE-AP, respectively. However, although wild-type MNV processed this boundary to completion, only partial cleavage was observed with the double domain swap chimaera or the MNV/SVD2/D54E construct. It is possible the difference in the P3 residue between these two boundaries influences cleavage efficiency although this is considered unlikely, given the minimal interaction the P3 residue has with the protease and that a previously described interaction in HNV protease between Lys^162^ and the P3 residue is likely to be conserved in MNV [25,26]. Alternatively, these differences could reflect a differential cleavage order and/or *cis*–*trans* cleavage requirements of MNV and HNV or the presence of different functional NS precursors in HNV. Both stable and/or transient NS protein precursors have been shown or suggested to have roles in the replication of several positive strand RNA viruses [46–49]. The NS5-encoded VPg, which acts as a primer for viral RNA replication, has been shown to be nucleotidylylated by NS7 in MNV and by NS7 and NS6NS7 in HNV [50–53]. Even though these studies have shown that VPg can be nucleotidylylated directly, it is unclear what the natural substrate for nucleotidylylation is in norovirus infection. In other positive strand RNA viruses, such, as picornaviruses, the 3BC and 3BCD precursors have been shown to be employed for uridylylation [54,55], and it is possible a similar precursor is the natural template for nucleotidylylation in HNV. However, much of what is understood regarding the order of ORF1 processing stems from *in vitro* studies with HNV and *in cellulo* studies with MNV, and the exact order of ORF1 processing for either norovirus and thus the precursors formed, has not yet been defined with a natural template *in cellulo*.

To date, none of the crystal structures of norovirus proteases in complex with boundary substrates or peptide mimics contained any residues on the P′ side, downstream of the cleavage junction. Alignment of the amino acid sequences on the P′ side of the boundary reveals no clear amino acid conservation beyond the preference for smaller residues (glycine or alanine) in the P1′ position and lysine, glycine, glutamine or proline in the P2′ position, suggesting the P’ side may have relatively little influence in dictating boundary cleavage. Indeed, a recent study with HNV protease showed that substitution of proline for glycine at the P2′ position resulted in a only a 3-fold decrease in cleavage efficiency [25]. Despite this, it is difficult to properly assess from the current crystal studies and biochemical data the interactions of the norovirus protease with the P′ boundary residues. Crystal structures of the related picornavirus and sapovirus protease suggest that some residues on the prime side of the boundaries have a role in dictating substrate cleavage [56–60]. In the context of the present study, it is possible that some of the boundary restrictions observed by swapping domain 1 of the protease were due to specific recognition between domain 1 of the protease and the prime side of the junction, as opposed to interactions between the two domains of the protease.

The lack of a robust cell culture system for HNV has severely limited the ability to dissect properties of the HNV NS6 protease in an eukaryotic background and within the context of a complete ORF1 polyprotein. In the present work, we have established a new system to study core functions of the NS6 protease. This system offers significant advantages over the current *in vitro* based assays and the few available replicon models in that it allows for assay of protease cleavage upon multiple boundaries in the context of a full ORF1 polyprotein, which is temporally expressed in mammalian cells in a transient system which does not require the laborious process of establishing stable replicon cell lines. The technological advancements offered by this new system, in combination with the current models, offers greater insights and a deeper understanding of the multi-functional nature of this apparently simple enzyme which has many complex interactions.

## Abbreviations

Ac: acetylated
HNV: human norovirus
MNV: murine norovirus
NS: non-structural
ORF: open reading frame
*p*NA: *p*-nitroaniline
SV: Southampton virus
